# Assigning a function to a conserved archaeal metallo-β-lactamase from *Haloferax volcanii*

**DOI:** 10.1007/s00792-012-0433-4

**Published:** 2012-02-18

**Authors:** Susan Fischer, Simona John von Freyend, Anice Sabag-Daigle, Charles J. Daniels, Thorsten Allers, Anita Marchfelder

**Affiliations:** 1 Biology II, University of Ulm, Albert-Einstein-Allee 11, 89069 Ulm, Germany; 2 Bio21 Molecular Science & Biotechnology Institute, The University of Melbourne, 30 Flemington Road, Parkville, VIC 3010 Australia; 3 Department of Microbiology, Ohio State University, 484 W. 12th Avenue, Columbus, 43210 USA; 4 School of Biology, Queen`s Medical Centre, University of Nottingham, Nottingham, NG7 2UH UK

**Keywords:** Metallo-β-lactamase, tRNase Z, *Haloferax volcanii*, PhnP, HVO_2763

## Abstract

The metallo-β-lactamase family of enzymes comprises a large group of proteins with diverse functions in the metabolism of the cell. Among others, this superfamily contains proteins which are involved in DNA and RNA metabolism, acting as nucleases in e.g. repair and maturation. Many proteins have been annotated in prokaryotic genomes as being potential metallo-β-lactamases, but very often the function has not been proven. The protein HVO_2763 from *Haloferax volcanii* is such a potential metallo-β-lactamase. HVO_2763 has sequence similarity to the metallo-β-lactamase tRNase Z, a tRNA 3′ processing endonuclease. Here, we report the characterisation of this metallo-β-lactamase HVO_2763 in the halophilic archaeon *Haloferax volcanii*. Using different in vitro assays with the recombinant HVO_2763, we could show that the protein does not have tRNA 3′ processing or exonuclease activity. According to transcriptome analyses of the HVO_2763 deletion strain, expression of proteins involved in membrane transport is downregulated in the mutant. Therefore, HVO_2763 might be involved directly or indirectly in membrane transport.

**Electronic supplementary material:**

The online version of this article (doi:10.1007/s00792-012-0433-4) contains supplementary material, which is available to authorized users.

## Introduction

**Fig. 1:**
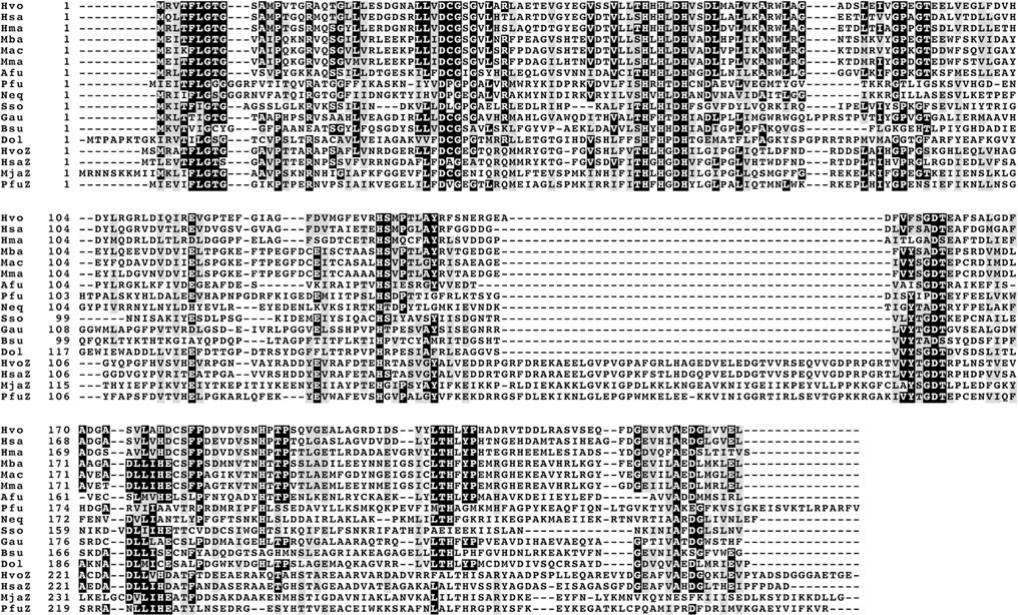
Alignment of NZ and tRNase Z proteins. NZ proteins and tRNase Z proteins from Archaea and bacteria are aligned revealing conserved amino acids (*black* identical amino acids, *grey* similar amino acids). The alignment clearly shows that the tRNase Z proteins all contain additional amino acids in the second half of the protein, the so called exosite (Vogel et al. 2005), which is not present in the NZ proteins. The exosite was shown to take part in substrate recognition (Vogel et al. 2005). NZ proteins and tRNase Z proteins (four bottom lines with Z: HvoZ, HsaZ, MjaZ and PfuZ) are shown from: Hvo, *H. volcanii*; Hsa, *Halobacterium salinarum*; Hma, *Haloarcula marismortui*; Mba, *Methanosarcina barkeri*; Mac, *Methanosarcina acetivorans*; Mma, *Methanosarcina mazei*; Afu, *Archaeoglobus fulgidus*; Pfu, *Pyrococcus furiosus*; Neq, *Nanoarchaeum equitans*; Sso, *Sulfolobus solfataricus*; Gau, *Gemmatimonas aurantiaca*; Bsu, *Bacillus subtilis;* Dol, *Desulfococcus oleovorans*; Mja, *Methanocaldococcus jannaschii*

Members of the metallo-β-lactamase (MBL) family have a broad substrate spectrum with most substrates comprising an ester bond and a negative charge. Among others, the MBL family includes class B β-lactamases, glyoxalases II, arylsulphatases, phosphodiesterases, phosphonate metabolism proteins PhnP and enzymes involved in the nucleic acid metabolism such as β-CASP and tRNase Z proteins (Dominski 2007; Späth et al. 2007). The MBL protein family is characterised by five conserved sequence motifs, including the histidine motif HxHxDH. These motifs facilitate a specific tertiary structure of the members of this family with two central β-sheets flanked by α-helices (αββα). Divalent cations like Zn^2+^, Fe^2+^ or Mn^2+^ are bound in the reactive center of the MBL enzymes and are essential for catalytic activity (Bebrone 2007; Wenzel et al. 2004). The β-CASP subfamily includes proteins that bind nucleic acids through the β-CASP domain and function primarily in DNA repair. Of the β-CASP proteins, only CPSF-73 acts as endonuclease on mRNA precursors to allow subsequent polyadenylation (Callebaut et al. 2002; Dominski 2007). The MBL enzyme tRNase Z generates the mature 3′ end of tRNA molecules by removal of the 3′ trailer of precursor tRNA in the three domains of life (Vogel et al. 2005). Recently, it was established that the tRNase Z enzyme of *Haloferax volcanii* also processes the 5′ end of the 5S rRNA precursors where a tRNA-like structure occurs (Hölzle et al. 2008). Apart from RNA precursors, tRNase Z can also process small chromogenic substrates like bpNPP (bis(*p*-nitrophenyl)phosphate) and TpNPP (thymidine-5′-monophosphate-*p*-nitrophenylester). Furthermore, the tRNase Z of *Pyrococcus furiosus* was shown to cleave the glyoxalase II substrate SLG (S-d-lactoylglutathione) (Späth et al. 2008). The protein exists in a short form (280–360 amino acids) and a long form (750–930 amino acids); the former is present in all three domains of life, whereas the latter is only found in eukaryotes (Späth et al. 2007). The N- and C-terminal domains of the long tRNase Z enzyme not only share sequence similarities, but also share structural motifs such as an intact His-motif in the C-terminal and a degenerated His-motif in the N-terminal part of the protein. Thus, the long enzyme version may have evolved out of two short tRNase Z proteins by gene duplication (Tavtigian et al. 2001). Another possibility for the evolution of the long tRNase Z form is the fusion of two distinct genes, the short tRNase Z and a gene with sequence similarity to the tRNase Z. A candidate gene (HVO_2763) for such a fusion event was found in the domain Archaea by BLASTP searches using the N-terminal portion of the eukaryotic tRNase Z as a query. This gene codes for a metallo-β-lactamase with unkown function and is present in most archaeal and some bacterial genomes (Fig. 1). This gene has recently been assigned a unique archaeal COG number (arCOG00500) distinguishing it from the closely related gene for tRNase Z in the updated 2009 arCOG database (Makarova et al. 2007). Due to its sequence similarity with the N-terminal part of the long tRNase Z, we named the protein HVO_2763 NZ protein (protein, homologous to the N-terminal part of the long tRNase Z). As additional functions for the long tRNase Z proteins were proposed by several authors (Dubrovsky et al. 2000; Smith and Levitan 2004; Takaku et al. 2003; Tavtigian et al. 2001), the functional investigation of the NZ protein as a potential fusion partner of the short tRNase Z might help to uncover these. The *H. volcanii* NZ protein (NZ, HVO_2763) is annotated as “metal dependent hydrolase” in the genome browser HaloLex (http://www.halolex.mpg.de/public/), and the corresponding gene is flanked by the genes coding for isopentenyl phosphate kinase (HVO_2762) and an ABC-type transport system permease protein with di-/oligopeptides as probable substrates (HVO_2764) (Supplementary Fig. 1). The *nz* gene appears to be monocistronic and comparisons of the *nz* gene environment in other closely related organisms did not indicate conservation of neighbouring genes.

To unravel the function of this metallo-β-lactamase, we used in vitro experiments and in vivo approaches.

## Materials and methods

### Strains and culture conditions

*H. volcanii* strains H26 (Δ*pyrE2*), H53 (Δ*pyrE2*, Δ*trpA*) (Allers et al. 2004) and Δ*nz* (Δ*pyrE2*, Δ*trpA*, Δ*nz::trpA* ^+^) were grown aerobically at 45°C in Hv-YPC medium or in Hv-Ca and Hv-Min medium using different supplements (for details see “Phenotypical analyses”) (http://www.haloarchaea.com/resources/halohandbook/Halohandbook_2008_v7.pdf).

*E. coli* strains DH5α (Invitrogen), GM121 and BL21-AI (Novagen) were grown aerobically at 37°C in 2YT and LB medium.

### Recombinant protein expression and purification

The sequence of the gene coding for the protein HVO_2763 was obtained from HaloLex. Chromosomal DNA from *H. volcanii* H53 was isolated by DNA spooling (http://www.haloarchaea.com/resources/halohandbook/Halohandbook_2008_v7.pdf) and the *nz* gene was amplified using primers hvonz1 (primer sequences are listed in Supplementary Table 1) and hvonz2 (containing an *Xho*I restriction site). The resulting PCR product was ligated with an *Sma*I digested vector pBluescriptII (Stratagene), yielding the plasmid pBlue-hvonz. The product from a second PCR with the template pBlue-hvonz and the oligonucleotides NZ-NEU (containing an *Nco*I restriction site) and RS using the Advantage-HF 2 PCR Kit (Clontech) was digested with *Nco*I and *Xho*I, and ligated with the similarly digested vectors pET29a (Novagen) and pET32a (Novagen). Further cloning of the *nz* gene sequence into the vectors pET28a (Novagen), pET45b (Novagen), pET51b (Novagen) and pTXB1 (Biolabs) was achieved by PCR with selected primers containing suited restriction sites (NZ-NDE28/NZ-SAC with *Nde*I/*Sac*I, NZ-PML45/NZ-SAC with *Pml*I/*Sac*I, NZ-KPN51/NZ-SAC with *Kpn*I/*Sac*I, NZ-NDE-XB1/NZ-XHO-XB1 with *Nde*I/*Xho*I) using pET32a-hvonz as a template. The *E. coli* strain BL21-AI (Novagen) was transformed with the resulting plasmids pET28a-hvonz, pET45b-hvonz, pET51b-hvonz, pTXB1-hvonz as well as pET29a-hvonz; the *nz* gene was expressed and the resulting recombinant NZ was purified according to the manufacturers’ protocol using S-protein agarose (pET29a-hvonz, Novagen), His-bind columns (pET28a-hvonz, pET45a-hvonz, Novagen), Strep-tactin superflow columns (pET51b-hvonz, Novagen) and Chitin resin (pTXB1-hvonz, Biolabs). The purified proteins were concentrated using Amicon Ultra-4 centrifugation units (10000 MWCO) (Millipore), dialysed against 20 mM Tris/HCl pH 7.5, 150 mM NaCl and stored at −20°C for in vitro activity tests. The purity of the expressed proteins was confirmed by SDS-gel electrophoresis.

For expression in *Haloferax*, the NZ reading frame was cloned downstream of the 3 × FLAG peptide cDNA into the pTA927 vector (Allers et al*.* 2010). *Haloferax* strain H26 was transformed with the plasmid, and subsequently a soluble protein extract from *Haloferax* was prepared. To isolate the protein extract, cells were grown to an OD_650_ = 0.9 in Hv-Ca broth including 1 mM tryptophan. One hour before harvest, the tryptophan was added to a final concentration of 3 mM. Cells were pelleted and the resulting pellet was washed twice with enriched PBS (2.5 M NaCl, 150 mM MgCl_2_, 1× PBS (137 mM NaCl, 2.7 mM KCl, 10 mM Na_2_PO_4_, 2 mM KHPO_4_, pH 7.4)) at 4°C and subsequently resuspended in lysis buffer (50 mM Tris/HCl pH 7.4, 1 mM EDTA, 150 mM NaCl). Cells were incubated on ice with slight shaking for 30 min, followed by sonification (5 × 30 s) on ice. After centrifugation (30000*g*, 15 min) the FLAG-NZ fusion protein was affinity purified from the lysate. For affinity purification, 1 ml of ANTI-FLAG M2 affinity gel (Sigma) was washed 5 times with ice-cold 10 ml washing buffer (50 mM Tris/HCl pH 7.4, 150 mM NaCl) before the lysate was added. After incubation overnight (14–16 h) at 4°C, ANTI-FLAG M2 affinity gel was washed eight times with ice-cold 10 ml washing buffer. The elution of the FLAG fusion protein was performed by the addition of 3 × FLAG peptide (150 ng/μl final concentration) to 4 ml washing buffer. The samples were incubated at 4°C for 30 min with gentle shaking. In a final elution step, the affinity gel was rinsed with 2 ml of washing buffer. The recombinant protein fraction was concentrated using Amicon Ultra-4 centrifugal filter units (10000 MWCO) (Millipore) and dialysed overnight against 50 mM Tris/HCl pH 7.4, 20 mM MgCl_2_ and 1 M KCl. The resulting protein fraction was analysed using SDS-PAGE.

**Fig. 2:**
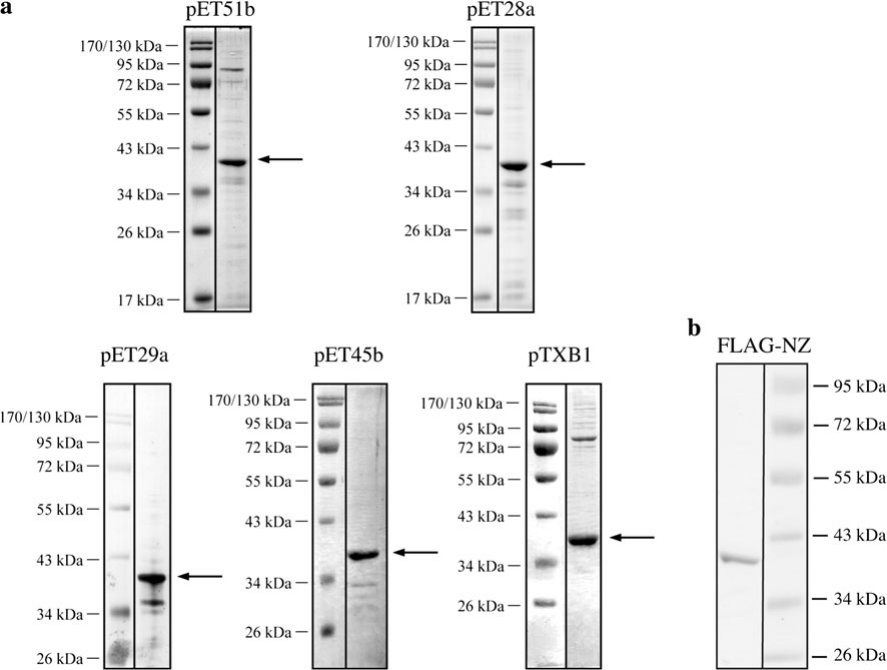
Recombinant NZ proteins with different tags are successfully purified from *E. coli* and *Haloferax*. **a** Expression in *E. coli*: SDS-PAGE of the different recombinant NZ proteins after purification; in each gel 1 μg protein was loaded. Above each gel, the plasmid type which was used for expression is stated (see also Supplementary Fig. 2). Gels were stained using Coomassie or silver and the protein size marker is given on the *left*. **b** Expression in *Haloferax*: SDS-PAGE of FLAG-NZ fusion protein after purification; 500 ng protein was loaded. The gel was stained using Coomassie and the protein size marker is given on the *right*

### In vitro activity tests

The recombinant proteins were tested for in vitro processing activity against *O. berteriana* pre-tRNA^Tyr^, *H. volcanii* pre-tRNA^Ala^ and *H. volcanii* pre-5S rRNA. These substrates were produced by in vitro transcription as described (Hölzle et al. 2008; Schierling et al. 2002). The in vitro processing assay was carried out with 500 ng NZ protein in a 100-μl reaction volume as described for HvoTrz in Späth et al. (2008). Recombinant HvoTrz (500 ng) was used as a positive control in the reaction. Further activity tests were performed with 1 μg of recombinant NZ protein in a 200-μl reaction volume using the appropriate buffers and substrates. Phosphodiesterase activity was measured using the substrates bis(*p*-nitrophenyl)phosphate (b*p*NPP) and thymidine-5′-monophosphate-*p*-nitrophenylester (T*p*NPP) with 20 mM of Tris/HCl pH 7.4, or sodium acetate pH 5.0, and 150 mM NaCl (Vogel et al. 2002). NaCl and KCl concentrations in the reaction buffer varied up to 500 mM. Glyoxalase II activity was determined as described in Vogel et al. (2002) using the substrate S-d-lactoylglutathione. Recombinant PfuTrz was utilised as a positive control (Späth et al. 2008). Phenole sulphatase activity was tested using the substrate *p*-nitrophenyl sulphate (*p*NPS) in two different reaction systems as published by Vogel et al. (2002) and Huggins and Smith (1947), respectively. Arylsulphatase A activity was determined with the substrates *p*-nitrocatechol sulphate (*p*NCS) and 4-methylumbelliferyl sulphate (MUS) as published by Vogel et al. (2002) and Chang et al. (1981), respectively. Alkaline phosphatase activity was measured using the substrate *p*-nitrophenyl phosphate (*p*NPP) as described (Vogel et al. 2002). The KCl concentration in the reaction buffers for the glyoxalase II, phenole sulphatase, arylsulphatase A and alkaline phosphatase activity tests varied from 0 to 2 M.

### Generation of a gene deletion

The deletion of the *nz* gene was achieved by using the pop-in/pop-out method as described previously (Allers et al. 2004, 2010; Bitan-Banin et al. 2003). The flanking regions of the *nz* gene were produced in two amplification reactions with primer pairs KONZ1/KONZ2 and KONZ3/KONZ4 on chromosomal DNA of *Haloferax* strain WFD11 (a kind gift of Prof. Dr. Felicitas Pfeifer, TU Darmstadt). The primers contained specific restriction sites for cloning: *Xho*I (KONZ1), *Sma*I (KONZ2, KONZ3), *BamH*I (KONZ4). The resulting PCR fragments of about 1000-nt length were subsequently cloned into the vector pBluescriptII (Stratagene), yielding pBlue-NZ3-4 and pBlue-NZ1-4. The tryptophan marker *trpA* was amplified using plasmid pTA132 (Allers et al. 2004) as template and oligonucleotides TRP1/TRP2. This maker was inserted into the *Sma*I digested plasmid pBlue-NZ1-4 producing pBlue-NZ1-4-TrpA. The insert NZ1-4-TrpA was then subcloned into the integrative vector pTA131 containing the *pyrE2* marker (Allers et al. 2004) yielding pTA131-NZ1-4-TrpA. After transformation of *H. volcanii* strain H53 with pTA131-NZ1-4-TrpA, the plasmid was integrated into the genome (pop-in). The subsequent selection for loss of the *pyrE2* marker by plating on 5-fluoro-orotic acid (5-FOA) revealed pop-out mutants. Chromosomal DNA was isolated from wild type and potential *nz* deletion mutants. Southern blot hybridisation was performed as described (Sambrook and Russell 2001) with the following modifications: 10 μg of *EcoR*V digested DNA was separated on 0.8% agarose gel and transferred to a nylon membrane (Hybond™-N, GE Healthcare). As hybridisation probe, the insert NZ3-4 was chosen and labelled using α-^32^P-dCTP and random prime kit Readiprime™II (GE Healthcare).

### Northern analyses

Total RNA was isolated from wild type and *nz* deletion mutants grown to exponential phase with the Illustra RNAspin Midi RNA Isolation Kit (GE Healthcare) or the modified Trizol RNA preparation protocol. For northern analysis, 5 or 10 μg RNA were denatured using glyoxal, separated on an agarose gel and blotted onto a nylon membrane (Hybond™-N, GE Healthcare). As hybridisation probe, the amplified *nz* gene was radioactively labelled using α-^32^P-dCTP and random prime kit Readiprime™II (GE Healthcare). For detection of the ABC-type sugar transport protein (permease) mRNA (HVO_A0146) and the glucan 1,4-α-glucosidase (HVO_A0149) mRNA DNA probes were generated using PCR with oligos ABC#1 and ABC#2 and Glucan#1 and Glucan#2, respectively. The resulting PCR products were labelled with a-^32^P-dCTP as described above.

### Phenotypical analyses

Growth of the *nz* deletion mutant was compared to the wild-type strain H53 in complex medium Hv-YPC and defined medium Hv-Min. Investigations were carried out both on solid and liquid media under aerobic conditions. The salt content of the complex medium ranged from 12 to 24% and different carbon sources were used with the Hv-Min medium (glucose, fructose, sucrose, glycerine, pyruvate, dl-lactate, trehalose, casamino acids, succinate/glycerine (10:1), dl-lactate/succinate/glycerine (11:10:1). Growth temperatures were changed from the optimal 45°C down to a minimum of 30°C or up to a maximum of 55°C. The growth after a limited exposure to 62°C (up to 7 h) was also examined. Cell shapes of *nz* deletion mutant and wild-type strain H53 were inspected using light and electron microscopy.

### *H. volcanii* custom tiled microarray and experimental design

Affymetrix was contracted to produce a custom tiled array for the *H. volcanii* DS2 strain recently sequenced at TIGR (Hartman et al. 2010) (Daniels et al., unpublished). The array is composed of 255984 perfect match (PM) probes, 25 nt in length, and a corresponding set of mismatch (MM) probes, all spaced in a 35-nt window. Ribosomal RNA operons, transposable elements, repeat sequences and sequences of the plasmid pHV2, which has been cured from the *H. volcanii* DS70 strain and its derivatives like H53 (Allers et al. 2004; Wendoloski et al. 2001), were omitted from the array. Based on the annotation of the *H. volcanii* DS2 genome, all coding regions and nearly all predicted intergenic regions greater than 50 nt in length are represented by at least one probe on each strand. Each array represents onefold coverage of the genome. Mapping of the probes to genomic positions and their positions within genes, as either coding (PP) or non-coding strand (PM), and to intergenic regions as either plus strand (PS) or minus strand (MS) was performed using BLASTN searches. The data reported in this work are median signal values (median gene expression values) for individual genes. The probe signal levels observed represent the combined effects of transcript synthesis and RNA turnover on the RNA population. However, we refer to these differences as changes in transcription of individual genes.

### RNA isolation

The modified Trizol RNA preparation protocol was used to isolate total cellular RNA from *H. volcanii* grown to OD_650_ = 0.1. Aliquots containing 30 μg of total RNA were provided to the Biomedical Genomics Core of The Research Institute at Nationwide Children’s Hospital, Columbus, Ohio (BGC-NWCH). Final quality checks of the RNA were performed using capillary electrophoresis.

### Array processing

All array processing was performed by the BGC-NWCH Research Institute. Since a standard protocol was not available for use with the *H. volcanii* custom array, and the *H. volcanii* genome has a high GC content (65%), the established protocol for the Affymetrix™ *Pseudomonas aeruginosa* (66% GC) gene array was tested with *H. volcanii* and found to give high signal response. Consequently, the *P.* *aeruginosa* protocol was adopted and used for all subsequent analyses. This protocol included cDNA synthesis, RNA removal, cDNA fragmentation, terminal labelling, hybridisation, washing and scanning of the array. Array data, as .cel files, were provided for further analysis and will be deposited at the National Center for Biotechnology and Information (NCBI) Gene Expression Omnibuss (GEO) (pending).

### Data analysis

To minimise the bias effects of technical variations between comparable arrays, quantile scaling (Auer et al. 2007) was applied to the combined data from a total of 16 array experiments, where each consisted of three independent arrays. Individual perfect match probes (PM) were adjusted for non-specific hybridisation by subtracting the signal from the corresponding mismatch probe (MM). Those probes giving a negative difference (MM > PM) were assigned a value of 1. Using these corrected values and the assignment of probes to individual genes and intergenic regions, median values were determined for probe sets that encompassed individual gene regions, both as sense (PP) and antisense strands (PM) and for all intergenic regions as both plus strand (ps) and minus strand (ms). These data are the reported expression values, EM. The log_2_ value for the ratio, EM experimental/EM control, was also determined for each gene and intergenic region. Wilcoxon signed-rank test was used to determine if the mean of the probe signals for individual genes or intergenic regions were significantly different when control and mutant strains were compared. For a confidence level of 95%, and approximately 4000 genes, a signed-rank value <10^−5^ was considered evidence for a significant difference between the experimental and control probe populations for a particular gene. A median expression level of ≥800 for an individual gene was considered as evidence that a gene was expressed, and defined as the minimum transcription level (MTL). This value represents the 75th percentile of the median expression value for all genes that exhibit twofold increased expression (log_2_ EM experimental/EM control). Genes or intergenic regions where the experimental or control EM values met the MTL (≥800), and whose signed-rank values for the pair were ≤10^−5^, were considered as showing significant changes in expression. All numerical calculations were performed using JMP^®^ (JMP^®^ 7.0.2).

## Results

### Expressing the *nz* gene in *E. coli* and *Haloferax*

In order to analyse the function of the so far uncharacterised NZ protein in vitro, we generated a recombinant NZ protein by heterologous expression of the *Haloferax* *nz* gene in *E. coli*. Different vectors and purification systems were used, resulting in five different recombinant proteins with various short tags (StrepII-tag, S-tag, His-tag and Intein-tag) (Supplementary Fig. 2). As shown previously for the tRNase Z and the Lsm protein from *H. volcanii* (Fischer et al. 2010; Späth et al. 2008), the *nz* gene of *H. volcanii* could be efficiently expressed as soluble protein in *E. coli* (Fig. 2a). The purity of the recombinant proteins was confirmed by SDS-gel electrophoresis (Fig. 2a). For a His-tagged (pET28a-hvonz) and the StrepII-tagged recombinant NZ protein, we tried to remove the tags by digesting them with thrombin and recombinant enterokinase, respectively. Unfortunately, it was not possible to remove the tags. However, the self-cleavage of the C-terminal intein-tag was successful, yielding a recombinant NZ protein with only four additional amino acids. For the following in vitro studies, the intein-derived NZ protein, as well as the four tagged recombinant NZ proteins, was used. In addition, the NZ protein was expressed in *Haloferax* as a FLAG fusion protein. Purification of the recombinant protein using FLAG agarose resulted in a pure recombinant protein fraction (Fig. 2b).

### The NZ protein is not active on RNA substrates

**Fig. 3:**
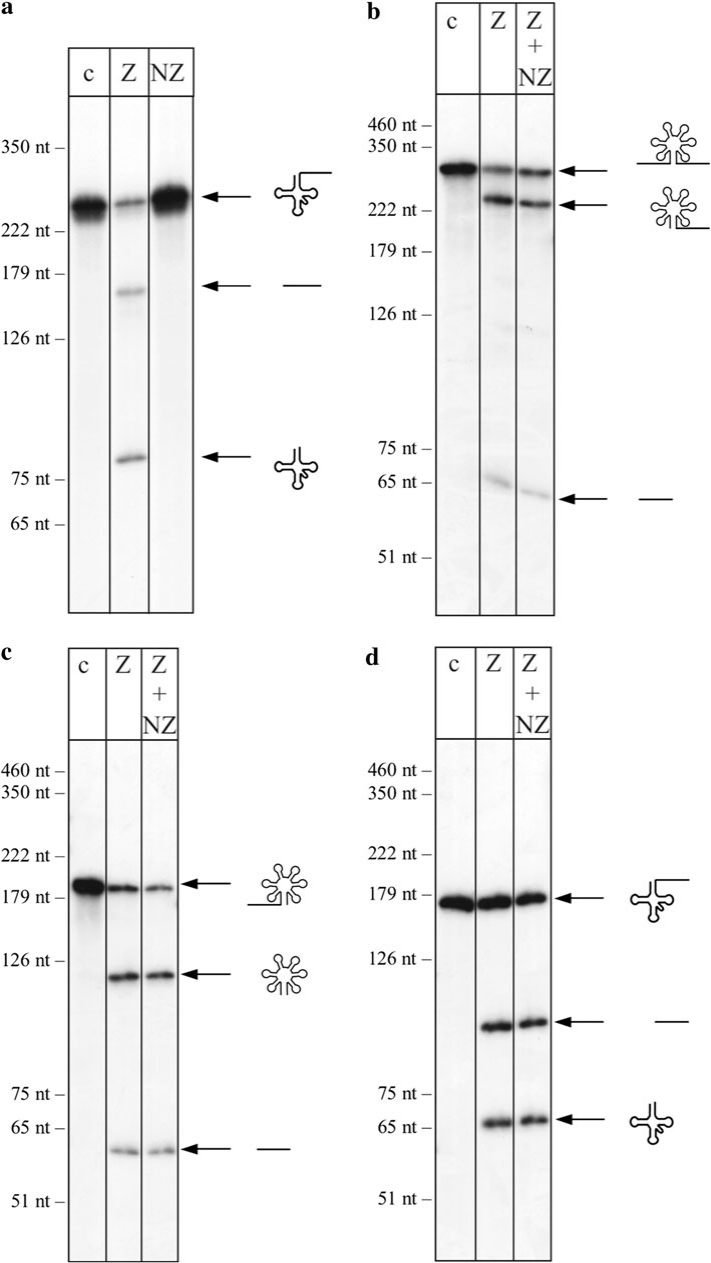
The NZ protein lacks RNA processing activity. tRNase Z substrates like pre-tRNA and pre-5S rRNA were incubated in vitro with recombinant NZ protein alone (*lanes NZ*), recombinant tRNase Z alone (*lanes Z*) or both tRNase Z and NZ (*lanes Z* *+* *NZ*) of *H. volcanii*. **a** pre-tRNA^Tyr^, **b** pre-5S rRNA with 5′ and 3′ extensions, **c** pre-5S rRNA with 5′ extension and **d** pre-tRNA^Ala^. Sizes of the DNA standard are given on the *left*, whereas precursors and products are shown schematically on the *right*. *Lanes c* control reaction without addition of proteins

To analyse whether the NZ protein can process RNA substrates, we incubated the recombinant protein expressed in *E. coli* with known tRNase Z substrates. Under the conditions tested, none of the substrates was cleaved (Fig. 3a). Neither tRNA nor 5S rRNA precursors that are efficiently cleaved by the *H. volcanii* tRNase Z (HvoTrz) enzyme in vitro are substrates for the tRNase Z homologue NZ (Fig. 3a, and data not shown). The RNA substrates were not affected at all by incubation with the NZ protein, showing that the enzyme also has no exonuclease activity. To investigate whether the NZ protein has any effect, e.g. an enhancing effect, on tRNA processing by the tRNA 3′ endonuclease tRNase Z, both recombinant proteins were incubated together with either pre-tRNA or pre-5S rRNA in in vitro processing assays. Addition of the NZ protein had no visible effect on the processing activity of tRNase Z (Fig. 3b–d). In addition, we incubated the NZ protein expressed in *Haloferax* with a pre-tRNA substrate, but again no processing could be observed (results not shown).

### Various chromogenic substrates are not converted by the NZ protein

BLAST searches showed that NZ has sequence similarities not only to tRNase Z, but also to some extent to other metallo-β-lactamases such as phosphodiesterase, glyoxalase II, phenole sulphatase, arylsulphatase and the alkaline phosphatase. To analyse, whether the *H. volcanii* NZ protein possesses these activities, the recombinant protein expressed in *E. coli* was tested in reactions with chromogenic substrates for each activity. A variety of processing conditions like low salt or high salt conditions were tested, but the NZ protein showed no reactivity towards any of the chromogenic substrates in vitro. In addition, the recombinant protein expressed in *Haloferax* was incubated with the phosphodiesterase substrate bpNPP, but no activity could be observed.

### An *nz* deletion mutant is viable and has wild-type-like growth

**Fig. 4:**
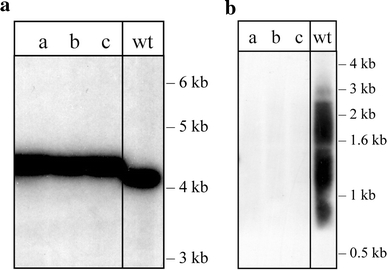
Deletion of the *nz* gene in *H. volcanii*. **a** Southern blot shows the absence of the *nz* gene in three independently generated *nz* deletion mutants. *Lanes a, b, c* *nz* deletion mutants, *lane wt* wild-type strain H53. Genomic DNA was digested using *EcoR*V resulting in fragment sizes of 4076 bp for the wild type and 4270 bp for the mutant. The blot was probed as described in “Materials and methods”. **b** Northern blot hybridisation confirms the absence of *nz* transcripts in the *nz* deletion mutants. *Lanes a, b, c* *nz* deletion mutants, *lane wt* wild-type strain H53. A DNA size marker is shown on the *right*

To confirm that the *nz* gene is indeed expressed in vivo, we used northern blot analyses (Fig. 4b) and RT-PCR (results not shown). The mRNA of the *nz* gene is clearly visible, and thus the gene is efficiently expressed. To examine the physiological role of the *H. volcanii* NZ protein, a deletion strain was constructed. The *nz* gene was deleted in this organism using the pop-in/pop-out method (Allers et al. 2004; Bitan-Banin et al. 2003) yielding the strain Δ*nz*. The complete removal of the *nz* gene was confirmed using a Southern blot (Fig. 4a). In addition, deletion of the *nz* gene correlated with the loss of the *nz* transcript in northern blot hybridisation (Fig. 4b) and RT-PCR (results not shown). The expression of the genes upstream and downstream of the *nz* gene was not affected by the gene deletion as shown by the transcriptome analysis (see below). The *nz* deletion mutant was viable under all conditions tested and no morphological changes were observed. The growth of the strain Δ*nz* did not vary from the wild type when salt concentration and carbon source in the media or growth temperature were changed (data not shown). A similar observation was made in *E. coli*, where the deletion of the tRNase Z gene also showed no phenotype (Schilling et al*.* 2004).

### The transcriptome of the *nz* deletion mutant changes significantly for transport-related genes

**Fig. 5:**
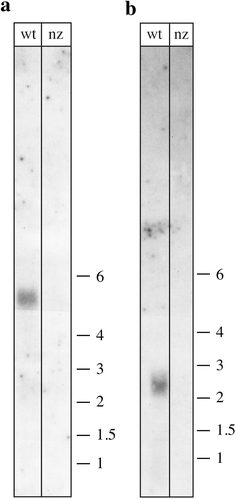
Confirmation of microarray data by northern analyses. To confirm the results obtained by the microarray experiments, we analysed the expression of two of the downregulated genes (see Table 1): HVO_A0146 and HVO_A0149 using northern blots. **a** Expression of the ABC-type sugar transport protein (permease) mRNA (HVO_A0146). The mRNA for HVO_A0146 is readily expressed in wild-type cells, whereas in the *nz* deletion strain the mRNA cannot be detected anymore. According to the northern blot, the mRNA is approximately 5.000 nt long, which suggests that the four genes HVO_A0145, HVO_A0146, HVO_A0147 and HVO_A0148 are expressed as one multicistronic mRNA (the open reading frames are altogether 4.7-kb long). **b** Expression of the glucan 1,4-α-glucosidase (HVO_A0149) mRNA. The mRNA for the HVO_A0149 gene is according to the expression in wild-type cells, approximately 2.400 nt long (the open reading frame of this mRNA is 2.2 kb). The gene is not expressed in the *nz* deletion strain. *Lanes wt* RNA isolated from wild-type cells, *lanes nz* RNA isolated from the *nz* deletion strain. The sizes of RNA markers in thousands of nucleotides are shown on the *right*

The transcriptome of the *H. volcanii* *nz* deletion mutant was investigated and compared to the wild type using a custom tiled microarray encompassing the *H. volcanii* genome. Three biological replicates of the wild type and the Δ*nz* strains were analysed and the transcriptional differences of at least twofold between the two strains are presented in Table 1. Genes where the experimental or control EM values met the MTL (≥800), and whose signed-rank values for the pair were ≤10^−5^, were considered as showing significant changes in expression. The transcriptome data clearly showed that the deletion of the *nz* gene did not affect the expression of the flanking genes. In the *nz* deletion mutant, only one transcript was upregulated with a median gene expression value above the MTL and a signed-rank value <10^−5^, namely *trpA*, the selectable marker which was used for generating the deletion, (Table 1, Supplementary Table 2). There were ten genes that exhibited at least a twofold decrease in expression in the *nz* mutant compared to the wild type. Of these ten genes, the majority encode transport-related proteins (Table 1). For example, HVO_2055 is annotated as a probable transport protein and is downregulated twofold in the *nz* deletion mutant (Table 1, Supplementary Table 3). A putative ABC-type transport system with zinc as the probable substrate (HVO_2397-2399) also exhibited downregulation in the *nz* deletion mutant compared to the isogenic wild-type strain (Table 1, Supplementary Table 3). The HVO_2397-2399 genes appear to be in an operon, and while not all the transcripts are changed up to twofold in the ∆*nz* strain, the signed-rank values indicate that the change in expression is well supported. A similar decrease in expression was found for the genes HVO_A0146 to HVO_A0148 annotated as an ABC-type sugar transport system (Table 1). To confirm the downregulation of those genes, we performed northern analyses for the ABC-type sugar transport protein (permease) mRNA (HVO_A0146) and the glucan 1,4-α-glucosidase (HVO_A0149) mRNA. The northern blots clearly confirm the downregulation as concluded from the microarray experiments (Fig. 5).

**Table 1:** Deletion of the *nz* gene causes significant changes in transcription levels of genes involved in transport mechanisms and central metabolism

GenBank no.	Protein function	EM (wild type)	EM (Δnz)	Signed rank	Log_2_ (EMΔ*nz*/EMwt)
Upregulated					
HVO_0789	Tryptophan synthase, α subunit	276.0	1445.0	9.55E−16	2.39
Downregulated					
HVO_A0146	ABC-type sugar transport protein (permease)	741.0	173.0	2.05E−11	−2.10
HVO_A0147	ABC-type sugar transport protein (permease)	837.0	270.0	2.11E−13	−1.64
HVO_A0149	Glucan 1,4-α-glucosidase	744.6	255.0	8.27E−22	−1.55
HVO_A0148	ABC-type sugar transport system, periplasmic substrate-binding protein	784.8	291.1	3.12E−17	−1.43
HVO_2401	Glycine cleavage system P-protein	3663.0	1363.0	2.42E−36	−1.43
HVO_2060	GDP-mannose mannosyl hydrolase	721.7	268.7	1.35E−06	−1.43
HVO_A0196	Hypothetical protein	719.0	299.0	8.42E−14	−1.27
HVO_2397	ABC-type zinc transport system, periplasmic substrate-binding protein	3012.0	1411.7	7.61E−28	−1.09
HVO_2055	Probable transport protein, putative	1112.8	526.4	2.66E−25	−1.08
HVO_2061	Dolichyl-P-glucose synthetase	733.2	356.6	1.97E−05	−1.04

Expression medians (EM) obtained with the tiled microarrays are listed for all transcripts with a log_2_ value >1 or <−1. Significance of the transcript upregulation in the Δ*nz* mutant compared to the wild type (wt) can be deduced from the signed-rank value. The GenBank no. corresponds to the putative ORF designation from the *H. volcanii* genome annotation. The gene HVO_0789 codes for the *trpA* marker that replaced the *nz* gene in the *nz* deletion mutant. This is the only transcript which is upregulated in the deletion mutant

## Discussion

To analyse the function of the metallo-β-lactamase NZ conserved in many Archaea and some bacteria, different in vitro and in vivo approaches were used. According to the results obtained, the NZ protein is not involved in RNA processing. Interestingly, deletion of the *nz* gene results in downregulation of proteins involved in membrane transport.

The in vitro tests with the different recombinant NZ proteins showed no activity towards any tRNA precursor under a variety of conditions tested. The NZ protein does not contain the exosite, which is important for substrate recognition for the tRNase Z enzymes (Vogel et al*.* 2005), and this might explain the lack of activity towards tRNA substrates. Also, other RNA precursors analysed were not substrates for this enzyme. Furthermore, substrates for other metallo-β-lactamases tested were also not processed. Since different methods of generating a recombinant NZ protein were employed and a variety of incubation conditions tested, the failure to process these substrates are very likely not due to the in vitro approach. Therefore, despite the sequence similarity to the tRNA 3′ processing endonuclease tRNase Z and other MBL enzymes, the protein NZ does not seem to be active in RNA cleavage or processing of other MBL substrates.

However, the transcriptome data and northern analyses clearly show that removal of the NZ protein results in downregulation of proteins involved in membrane transport. The MBL protein PhnP from *E. coli* is encoded in the *phn* operon as part of an ABC transport and utilisation system for phosphonate (Hove-Jensen et al. 2010) and thereby also linked to transport processes. In addition, an MBL cistron was found within an ABC maltose transporter operon in *Lactobacillus casei*, suggesting a possible but not yet characterised role of this metallo-β-lactamase protein in maltose transport (Monedero et al. 2008). The genes HVO_A0146-A0148 coding for an ABC sugar transport machinery were found to be downregulated at least twofold or more in the *nz* deletion strain (see Table 1). A possible connection of the NZ protein to this ABC sugar transport system might suggest a function of NZ in the metabolism of the transported sugars, comparable to the suggested role of the PhnP protein in the metabolism of phosphonate (Hove-Jensen et al. 2010). One protein component of this transport system (HVO_A0147) was tentatively annotated as permease specific for the trehalose/maltose transport based on trehalose/maltose ABC-system permease of e.g. *Pyrococcus furiosus* and *Thermococcus litoralis* (http://halo4.umbi.umd.edu/cgi-bin/haloweb/hvo.pl?operation=gene_table&id=140&line_per_page = 20&table_type = replicon&group_name = pHVo400 ). No other transporter responsible for trehalose transport could be found in the *Haloferax* genome as annotated at NCBI.

A possible role of NZ in the regulation of a potential trehalose/maltose transporter might also suggest that the NZ protein participates in the osmotolerance of the *Haloferax* cell. Although the main part of the osmoprotection of *H. volcanii* in high salt environments is provided by high intracellular concentrations of potassium ions, metabolomic analyses show that also organic compounds like trehalose are found in substantial amounts (>5 μmol/g dry weight) in *H. volcanii* (M. Kucklick, personal communication). Thus, NZ could be involved in osmoprotection by regulating the cellular concentrations of trehalose and glycine via the trehalose metabolism and regulation of the glycine cleavage system (see Table 1). The downregulation of the HVO_2048 gene transcript, which is likely to be encoding the reversible trehalose synthase of the TreT pathway (Prof. Dr. Bettina Siebers, personal communication), to 65% of wild-type level might confirm this hypothesis (see Supplementary Table 3). But to show an involvement of NZ in osmotolerance, data are required to prove that function.

Another ABC-type transporter clearly influenced by the NZ protein is the transporter system encoded in the operon HVO_2397-2399, with the annotated probable substrate being zinc. Here, a significant decrease, to about 50% of the wild-type level, was observed for the transcripts of all three cistrons in the *nz* deletion mutant (Tables 1, Supplementary Table 3).

Interestingly, the NZ protein is encoded on the genome next to two proteins which are involved in transport: two ABC-type transport system permease proteins with di-/oligopeptides as probable substrates (HVO_2764 and HVO_2765) (Supplementary Fig. 1). This might be another indicator that NZ is as well involved in transport. But for a final statement that NZ has a function in transport, additional data are required.

## Electronic supplementary material

Below is the link to the electronic supplementary material.

Supplementary material 1 (PDF 502 kb)
