# Supplementary material for: Assigning a function to a conserved archaeal metallo-β-lactamase from Haloferax volcanii
Source: Extremophiles. 2012 Feb 18;16(2):333–43. doi: 10.1007/s00792-012-0433-4 (PMC3296008; doi:10.1007/s00792-012-0433-4)
Supplement: Supplementary file 1 — Supplementary material 1 (PDF 502 kb) [file 792_2012_433_MOESM1_ESM.pdf]

Supplementary Table 1. Primer sequences.

Sequences of oligonucleotides used. Restriction endonuclease sites used in cloning are underlined.

Primer	Sequence (5' – 3')
hvonz1	TAATACCATGGCATGCGCGTCACGTTCCTCGGAAC
hvonz2	TAATATCTCGAGTCAGAGTTCGACGACCAACCCG
NZ-NEU	GGATCCCCCTAATACCATGGCTATGCGCGTCACGTTCCTCGG
NZ-NDE28	GGATCCCCCTAATACATATGATGCGCGTCACGTTCCTCGGAAC
NZ-SAC	GGATCCCCCTAATAGAGCTCTCAGAGTTCGACGACCAACCCG
NZ-PML45	GGATCCCCCTAATACACGTGATGCGCGTCACGTTCCTCGGAAC
NZ-KPN51	GGATCCCCCTAATAGGTACCGATGCGCGTCACGTTCCTCGG
NZ-NDE-XB1	GGATCCCCCTAATACATATGCGCGTCACGTTCCTCGGAACCGG
NZ-XHO-XB1	GGATCCCCCTAATACTCGAGTTCGACGACCAACCCGTCTTCTG
KONZ1	TAATATCTCGAGCGCGACGACCATCGTCATCC
KONZ2	TAATATCCCGGGGCGCCGAGATACCACGGCGG
KONZ3	TAATATCCCGGGGTCTGACTCCGAGTCTGAATC
KONZ4	TAATATGGATCCGCTGTATCGTCTCCTTGGAC
TRP1	GGATCCCGTGGATAAAACC
TRP2	GAGCAGTTATGTGCGTTCCGG
ABC#1	GCTTCGTGCTAGCCACCACGAC
ABC#2	CGACCACTTGGACGCTTCGGGC
Glucan#1	GGGATTCGGCGGCATCGTGTCCTCACG
Glucan#2	GCGACTCTCCTCGTACTCTTCGGACG

Supplementary Table 2. Changes in the transcriptome I.

Up-regulated genes in the *nz* deletion mutant. Expression medians (EM) obtained with the tiled microarrays are listed for all transcripts with a $\log_2$ value > 0.5. Significance of the transcript up-regulation in the Δ*nz* mutant compared to the wild type (wt) can be deduced from the signed-rank value. For the protein products of the transcripts the pI and the number of transmembrane domains are given. Gene HVO_0789 encodes the tryptophan synthase and was used as marker gene to replace the *nz* gene. It is not present in the wildtype and therefore only expressed in the mutant strain.

Function/Functional class	GenBank no.	EM (Δnz)	EM (wildtype)	$\log_2$ (EMΔnz/EMwt)	Signed rank	pI/ TMD
**1. Transporter**						
ABC-type sugar transport system, periplasmic substrate-binding protein	HVO_2695	3347.7	2337.6	0.52	2.74E-15	4.3/0
**2. Diverse functions**						
tryptophan synthase, α subunit	HVO_0789	1445.1	276.0	2.39	9.55E-16	4.1/0

Supplementary Table 3. Changes in the transcriptome II.

Down-regulated genes in the *nz* deletion mutant. Expression medians (EM) obtained with the tiled microarrays are listed for all transcripts with a $\log_2$ value < –0.5. Significance of the transcript down-regulation in the Δ*nz* mutant compared to the wild type (wt) can be deduced from the signed-rank value. For the protein products of the transcripts the pI and the number of transmembrane domains are given.

Function/Functional class	GenBank no.	EM (Δ*nz*)	EM (wild type)	$\log_2$ (EMΔ*nz*/EMwt)	Signed rank	pI/ TMD
**1. Transporter**						
ABC-type sugar transport protein (permease)	HVO_A0146	172.7	740.7	-2.10	2.05E-11	5.7/6
ABC-type sugar transport protein (permease)	HVO_A0147	269.6	837.5	-1.64	2.11E-13	4.9/7
ABC-type sugar transport system, periplasmic substrate-binding protein	HVO_A0148	291.1	784.8	-1.43	3.12E-17	4.3/0
ABC-type zinc transport system, periplasmic substrate-binding protein	HVO_2397	1411.7	3012.0	-1.09	7.61E-28	4.4/0
probable transport protein, putative	HVO_2055	526.4	1112.8	-1.08	2.66E-25	9.1/14
ABC-type zinc transport system (permease)	HVO_2399	720.3	1429.8	-0.99	1.19E-15	4.4/9
Tat (twin-arginine translocation) pathway signal sequence domain protein	HVO_C0054	401.4	755.4	-0.91	2.34E-06	4.6/0
ABC-type zinc transport system, ATP-binding protein	HVO_2398	927.3	1701.3	-0.88	2.46E-13	4.5/0
Tat (twin-arginine translocation) pathway signal sequence domain protein	HVO_A0133	1918.9	3344.4	-0.80	1.83E-15	5/0
ABC-type phosphate transport system, periplasmic substrate-binding protein	HVO_2375	775	1192.4	-0.62	3.98E-05	3.8/0
ABC-type iron-III transport system, periplasmic substrate-binding protein	HVO_B0144	1952.9	1030.1	-0.52	2.89E-08	4.2/0
**2. Diverse functions**						
glucan 1.4-alpha-glucosidase	HVO_A0149	255.0	744.6	-1.55	8.27E-22	4.6/0
glycine cleavage system P-protein	HVO_2401	1363.2	3662.9	-1.43	2.42E-36	4.4/0
GDP-mannose mannosyl hydrolase	HVO_2060	268.7	721.7	-1.43	1.35E-06	4.3/0
dolichyl-P-glucose synthetase	HVO_2061	356.6	733.2	-1.04	1.97E-05	4.4/4
UDP-glucose 4-epimerase	HVO_2040	701.8	1371	-0.97	1.03E-10	4.4/0
sugar nucleotidyltransferase	HVO_2057	896.6	1689.7	0.91	9.50E-12	4.3/0
glutaredoxin-like protein	HVO_2396	4126.5	7706.7	-0.90	5.23E-06	4.4/0
Muc19 precursor, putative	HVO_2160	1055.3	1927.7	-0.87	1.29E-49	3.7/1
aminomethyltransferase (glycine cleavage system protein T)	HVO_2404	794.6	1408.6	-0.83	4.68E-07	4.1/0
proteinase IV-like protein	HVO_1987	614.8	1056.8	-0.78	8.75E-10	4.5/0
mannosyltransferase B, putative	HVO_2048	725.8	1110.6	-0.61	2.14E-09	5.1/0
N-acetylgalactosamine-4-sulfatase	HVO_2046	560.8	852.2	-0.60	2.09E-06	4.8/0
spore coat polysaccharide synthesis spsK	HVO_2056	3040.9	4477.4	-0.56	1.32E-07	4.4/0
UDP-glucose 4-epimerase	HVO_2059	669.2	982.1	-0.55	1.60E-06	4.2/0
lipopolysaccharide transferase family protein	HVO_2053	693.7	985.3	-0.51	4.12E-05	4.5/0
**3. Unknown functions**						
hypothetical protein	HVO_A0196	298.8	719.4	-1.27	8.42E-14	3.9/0
hypothetical protein	HVO_2047	388.6	717	-0.88	4.33E-08	4.8/0
hypothetical protein	HVO_2015	990.8	1718.8	-0.79	8.17E-09	4.1/0
hypothetical protein	HVO_A0432	1248.5	2035.8	-0.71	9.62E-05	5.1/0
hypothetical protein	HVO_C0025	1039.8	1596.4	-0.62	5.55E-07	4.1/0

Supplementary Figure 1. Genomic Location of the *nz* gene.

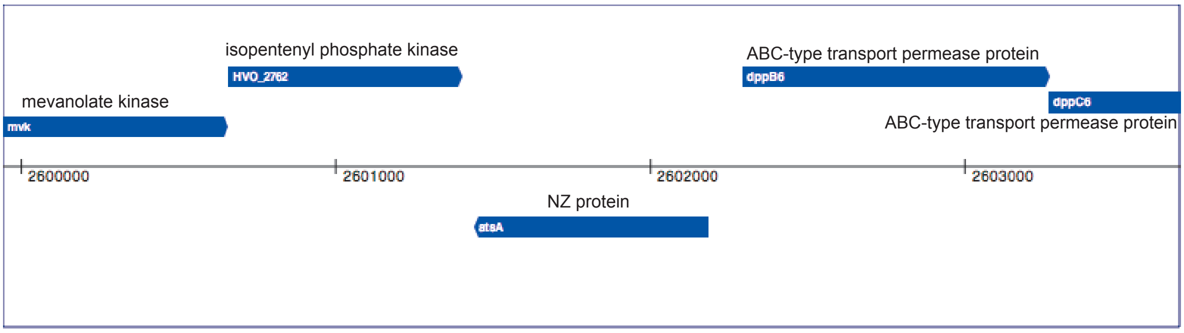

The *nz* gene is flanked by a gene coding for a kinase and an ABC-type transport permease protein. Both flanking genes are located on the other strand.

Supplementary Figure 2. Expression of recombinant NZ proteins with different tags in *E. coli*.

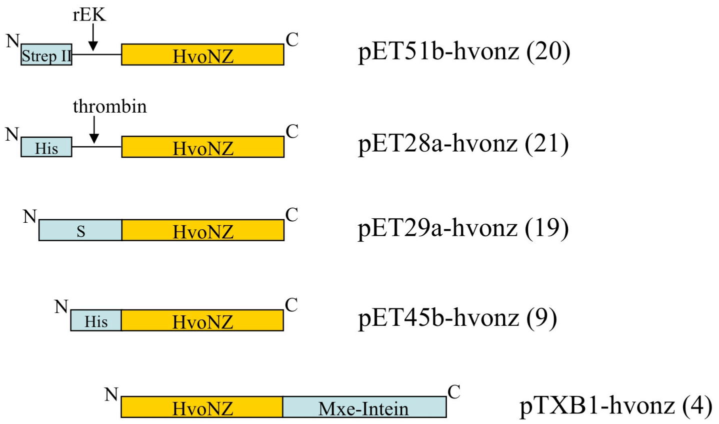

Schematic drawings of the NZ fusion proteins are shown. The NZ protein of *H. volcanii* was fused with different tag sequences at the N- or C-terminus. The number of additional amino acids of the purified proteins is given in brackets. Protease recognition sites are indicated by arrows.
